# Lycopene Mitigates Rat Liver Damage Induced by Lipopolysaccharide via Mechanisms Involving Oxidative Stress, Inflammation, and Apoptosis

**DOI:** 10.3390/cimb47110914

**Published:** 2025-11-04

**Authors:** Snežana Tešić Rajković, Andrija Rančić, Marko Stojanović, Jelena Živadinović, Ivana Ramić, Milica Nestorović, Sava Spasić, Elena Stanković, Ivan Nagorni, Vesna Brzački, Ilija Ilić, Miloš Dičić, Dušan Sokolović

**Affiliations:** 1 Faculty of Medicine, University of Niš, 18000 Niš, Serbia; snezanatesic@yahoo.com (S.T.R.); jelena5491@gmail.com (J.Ž.); milica20@yahoo.com (M.N.); brzackiv@gmail.com (V.B.); dicic@hotmail.com (M.D.); 2 Clinic for Gastroenterology and Hepatology, University Clinical Center Niš, 18000 Niš, Serbia; andrija.m.rancic@gmail.com (A.R.); marcss994@gmail.com (M.S.); drivanaramic93@gmail.com (I.R.); savaspasi@gmail.com (S.S.); stankovicelena008@gmail.com (E.S.) ivannagorni@gmail.com (I.N.); ilijailic.med@gmail.com (I.I.); 3 Clinic for Anesthesiology and Intensive Care, University Clinical Center Niš, 18000 Niš, Serbia; 4 Department for Surgery, University Clinical Center Niš, 18000 Niš, Serbia; 5 Institute for Treatment and Rehabilitation, Niška Banja, 18000 Niš, Serbia

**Keywords:** lipopolysaccharide, liver, lycopene, oxidative stress, inflammation, NF-κB

## Abstract

Background: Sepsis is a leading cause of mortality in intensive care units, with liver dysfunction representing a critical determinant of poor outcome, mainly associated with excessive inflammation and oxidative stress. Lycopene, a carotenoid with potent antioxidant and anti-inflammatory properties, has been proposed as a potential therapeutic agent. This study investigated whether lycopene supplementation mitigates lipopolysaccharide-induced oxidative and inflammatory liver injury in rats. Methods: Male Wistar rats, divided into four groups, were exposed to either lipopolysaccharide or a combination of lipopolysaccharide (10 mg/kg) and lycopene (6 mg/kg). In order to assess liver damage induced by lipopolysaccharide, hepatocellular injury markers, oxidative stress indices, nitric oxide metabolism, glutathione redox status, apoptotic enzyme activity, and inflammatory mediators were assessed in serum and liver tissue. Results: Lipopolysaccharide induced marked hepatocellular damage, characterized by elevated serum liver-cell damage parameters, and liver tissue xanthine oxidase, myeloperoxidase, thiobrabituric reactive substances, protein carbonyl content, deoxyribonuclease I/II activity, nuclear factor kappa B, tumor necrosis factor-α, and interleukin-6, alongside depletion of reduced glutathione and reduced glutathione reductase and glutathione peroxidase activities. Lyc pretreatment significantly attenuated liver enzyme leakage, oxidative damage, and cytokine release while restoring reduced glutathione and glutathione reductase activity. In contrast, lycopene had limited effects on glutathione peroxidase activity, nitric oxide/inducible nitric oxide synthase signaling, and nuclear factor erythroid 2-related factor 2 expression. Conclusions: These findings demonstrate that lycopene confers partial hepatoprotection in endotoxemic rats, primarily through suppression of oxidative damage and nuclear factor kappa B-mediated inflammation. Further studies are needed to clarify tissue-specific mechanisms and optimize dosing strategies in order to increase the efficacy of this carotenoid.

## 1. Introduction

Sepsis is a severe and often fatal clinical syndrome characterized by systemic inflammatory response and multiple organ dysfunction caused by an overwhelming immune reaction to infection [1]. Among the most common and studied inducers of sepsis in experimental models is lipopolysaccharide (LPS), a structural component of the outer membrane of Gram-negative bacteria, also known as endotoxin [2]. Upon release into the host circulation, either during bacterial lysis or infection, LPS triggers a potent inflammatory response primarily by activating Toll-like receptor 4 (TLR4) on immune and parenchymal cells, especially Kupffer cells and liver sinusoidal endothelial cells [3,4]. The liver, as a central immunologic and metabolic organ, plays a crucial role in clearing circulating LPS and bacteria, making it particularly susceptible to inflammatory damage during endotoxemia [5,6].

Hepatic immune cells, including Kupffer cells, neutrophils, and hepatic stellate cells, coordinate a complex response involving the release of cytokines, chemokines, and reactive oxygen species (ROS) [7]. While this response is initially aimed at neutralizing the infectious threat, excessive or uncontrolled activation can lead to oxidative stress, mitochondrial dysfunction, and hepatocellular injury [8]. One of the hallmark features of LPS-induced liver damage is oxidative stress, resulting from the excessive generation of ROS and reactive nitrogen species (RNS), including peroxynitrite. These species initiate lipid peroxidation, protein nitration, and deoxyribonucleic acid (DNA) damage, leading to impaired mitochondrial function and activation of cell death pathways. This interaction between LPS and immune system cells initiates a cascade of intracellular events, prominently involving the activation of the nuclear factor kappa B (NF-κB) pathway and subsequent upregulation of proinflammatory mediators such as tumor necrosis factor-alpha (TNF-α) and interleukin-6 (IL-6) [9].

Lycopene (Lyc), a fat-soluble molecule composed of eight isoprene units, belongs to a class of carotenoids that are present in various red fruits and vegetables [10]. Its plasma concentration after intake is debatable and arguably low; thus, it is suggested that its efficacy directly correlates with its concentration or the concentration of its metabolites [11]. The health benefits and health-promoting potential of Lyc have been proven previously in both animal and human studies [11,12,13]. Lyc is suggested to suppress chronic inflammation, such as that seen in ulcerative colitis [13], prevent the progression of atherosclerosis, and modulate obesity and liver fat deposition [11]. The majority of Lyc action is attributed to its ability to scavenge and interact with various ROS (e.g., quenching singlet oxygen) and thus prevent cell, cellular structure, and DNA damage [11]. The mechanisms through which Lyc acts with ROS, partially due to open-chain unsaturated structure, involve different changes in structure, which finally, through interaction with solvent, liberate thermal energy [11]. At the level of liver tissue, Lyc impacts not just ROS formation and consequences of its increase (lipid peroxidation and DNA damage) but also affects lipid metabolism enzymes, antioxidant enzymes (catalase (CAT) and superoxide dismutase (SOD), general metabolism (Krebs cycle), and inflammation [14].

Given the role of oxidative stress and inflammation in LPS-induced hepatic injury, the present study aims to evaluate the protective effects of Lyc in a rat model of LPS-induced sepsis and consequential liver damage. The investigation focuses on different biochemical markers of oxidative damage, nitric oxide signaling, glutathione metabolism, apoptosis, proinflammatory cytokines (particularly TNF-α and IL-6), and activation of the NF-κB and Nrf-2 pathways.

## 2. Materials and Methods

### 2.1. Chemicals

Lipopolysaccharide (LPS, *Escherichia coli* O111:B4) and lycopene (purity ≥ 90%) were obtained from Sigma-Aldrich (St. Louis, MO, USA). Lycopene was dissolved in corn oil immediately before administration. All other reagents were of analytical grade.

### 2.2. Animals and Experimental Design

Healthy male Wistar albino rats (7–8 weeks old, 175–225 g) were housed under controlled laboratory conditions (22 ± 2 °C, 50 ± 5% relative humidity, 12 h light/dark cycle) with ad libitum access to standard chow and water. The animals were obtained from the Vivarium of the Institute of Biomedical Research, the Faculty of Medicine, the University of Niš, Serbia. The study protocol was approved by the Institutional Animal Ethics Committee and conducted in accordance with EU Directive 2010/63/EU and the Guide for the Care and Use of Laboratory Animals (8th edition), as well as those provided by the laws of the Republic of Serbia (decision number 323-07-01762/2021-01, provided on 13 May 2021 by the Republic of Serbia Ethics committee for animals).

Rats were randomly divided into four groups (n = 6):

1. Control—single oral dose of vehicle (corn oil, p.o.);
2. Lycopene—single oral dose of lycopene (6 mg/kg);
3. LPS—single intraperitoneal injection of LPS (10 mg/kg in saline) [3,15];
4. LPS + Lycopene—single p.o. dose of lycopene (6 mg/kg) dissolved in corn oil [16], followed by a single intraperitoneal injection of LPS (10 mg/kg).

Animals were sacrificed under ketamine anesthesia 12 h after LPS injection. Blood was collected via cardiac puncture for serum analyses, and livers were excised, rinsed in ice-cold saline, blotted, and processed for biochemical assays. Liver tissue homogenates (10% *w*/*v*) were prepared in ice-cold buffer and centrifuged (5000× *g*, 15 min, 4 °C). The protein concentration was determined by Lowry’s method [17] using bovine serum albumin as a standard.

### 2.3. Serum Biochemical Analysis

After clotting at room temperature, the blood samples obtained from animals were centrifuged at 1500 rpm for 15 min at 4 °C. Serum alanine aminotransferase (ALT) and aspartate aminotransferase (AST) activities were measured using an automated biochemical analyzer (Olympus AU680, Olympus, Tokyo, Japan) with manufacturer-supplied reagents. Activities were expressed in U/L.

### 2.4. Tissue Biochemical Analyses

#### 2.4.1. Determination of ROS Generators

Xanthine oxidase (XO) activity was determined by quantifying uric acid formation through the oxidation of xanthine. The reaction was initiated by adding 0.6 mM xanthine as substrate, and the increase in absorbance was monitored spectrophotometrically at 293 nm [18]. Enzyme activity was calculated using the molar extinction coefficient of uric acid (ε = 12.6 mM^−1^ cm^−1^). Results were expressed as units per gram of tissue protein (U/g), where one unit corresponds to the amount of enzyme required to produce 1 μmol of uric acid per minute under assay conditions.

Myeloperoxidase (MPO) activity in liver tissue was determined using a colorimetric assay based on the oxidation of ortho-phenylenediamine (OPD) by hydrogen peroxide (H_2_O_2_). The reaction mixture, containing OPD, H_2_O_2_, citrate buffer (pH 5.0), and liver homogenate, was incubated at 25 °C for 5 min. After this time, the reaction was terminated by the addition of sulfuric acid. Absorbance was measured at 540 nm, and MPO activity was calculated as the optical density per milligram of protein (OD/mg of protein) [19].

#### 2.4.2. Lipid and Protein Oxidative Damage Determination

Lipid peroxidation in liver homogenates was determined as thiobarbituric acid reactive substances (TBARS) according to a previously described method [20]. Briefly, the homogenate was mixed with the thiobarbituric acid reagent and heated in a boiling water bath for 15 min. After cooling, the absorbance of the pink chromogen was measured at 532 nm. The TBARS concentration was calculated using the molar extinction coefficient 1.56 × 10^5^ M^−1^ cm^−1^ and expressed as nmol/mg protein.

Protein carbonyl content (PCC) levels were measured spectrophotometrically by incubating liver homogenate with 2,4-dinitrophenylhydrazine for derivatization of carbonyl groups [21]. The absorbance was measured at 370 nm, and concentrations were calculated using the molar extinction coefficient of DNPH (22 × 10^3^ L mol^−1^ cm^−1^), expressed as μmol/mg of protein.

#### 2.4.3. Antioxidant Enzymes Determination

Liver tissue CAT activity was determined by measuring the reaction absorbance at 405 nm following a reaction between tissue homogenate, H_2_O_2_ (substrate), and ammonium molybdate [21]. Enzyme activity was expressed as U/mg of liver tissue proteins.

The activity of SOD in liver tissue homogenates was determined using a colorimetric assay kit (Abcam653454) and previous methods [22]. The method is based on the reaction between water-soluble tetrazolium salt (WST) and superoxide anions in tissue homogenate, which was monitored at 450 nm. The activity was expressed as a % decrease in activity, according to the manufacturer’s instructions.

#### 2.4.4. Arginine/Nitric Oxyde Cycle Parameters Determination

NO production was estimated by measuring nitrite and nitrate levels via the Griess reaction. After deproteinization, equal volumes of liver tissue homogenates were incubated with sulfanilamide and N-(1-naphthyl)ethylenediamine dihydrochloride, and the absorbance was measured at 492 nm afterwards [23]. Concentrations were expressed as nmol/mg protein.

Arginase activity was determined by measuring urea formation from L-arginine. Following incubation with substrate, the reaction was stopped, and urea was quantified colorimetrically at 515 nm [24]. Activity was expressed as μmol urea/min/mg protein.

The L-citrulline concentration was measured using the diacetyl monooxime and thiosemicarbazide method [25]. Reaction mixtures were boiled for 5 min, cooled, and the absorbance was measured at 530 nm. Concentrations were expressed as μmol/mg protein.

The iNOS content was estimated using an ELISA (CUSBIO, CSB-E08325r, Houston, TX, USA) sandwich enzyme-linked immunosorbent assay kit. The obtained results are provided as IU/mg of tissue proteins.

#### 2.4.5. Determination of Reduced Glutathione (GSH) and GSH-Related Enzymes

The concentrations of reduced glutathione (GSH) were measured according to the previously described method [26]. Briefly, after deproteination of the supernatant from the liver tissue homogenate, DNTB reagent was added, and the absorbance of the mixture was measured at 410 nm. The final amount of GSH was expressed as nmol/mg of protein based on the standard curve constructed using GSH.

Glutathione reductase (GR) activity in the liver tissue was evaluated by tracking the reduction of GSSG to GSH in the presence of NADPH [27]. The absorbance of the solution was measured at 412 nm, and the results were expressed in nmol/mg of liver tissue protein.

The activity of glutathione peroxidase (GPx) was determined in the reaction mixture containing liver tissue homogenate, H_2_O_2_, and exogenously added GSH [26]. After the incubation, the amount of remaining GSH was determined in its reaction with DTNB and quantified using the standard curve.

#### 2.4.6. Determination of Alkaline-Dnase I and Acid-DNase II Activity

Methods for measuring liver tissue alkaline and acid DNase activity were performed using DNA as a substrate [27]. The activity of alkaline DNase I was determined at optimum pH = 7.4 using Tris-HCl buffer and Mg^2+^ ions as activators. In the case of acidic DNase II activity, the acetate buffer at optimum pH = 5.0 was used for the assay. The activity of the enzyme was expressed as IU/g of protein.

#### 2.4.7. Inflammation Mediators’ Determination

The determinations of liver tissue NF-κB level (NF-kappa-B-activating protein ELISA Kit, Wuhan Fine Biotech, Wuhan, China; ER0510), IL-6 (Quantikine ELISA Rat IL-6, R&DSystems, Minneapolis, MN, USA; R6000B), and TNF-α (Rat TNF alpha ELISA Kit Abcam, Boston, MA, USA; ab236712) were performed according to the manufacturer’s instructions. The amounts of NF-κB, IL-6, and TNF-α are provided as pg per mg of protein.

#### 2.4.8. Nuclear Factor Erythroid 2-Related Factor 2 (NRF-2) Content Determination

Liver tissue Nrf-2 content was determined using an ELISA assay kit (Abcam, Cambridge, MA, USA). The procedure was performed by following the manufacturer’s instructions and previous studies [28]. The obtained results are presented as pg of Nrf-2 per mg of liver tissue proteins.

### 2.5. Statistical Analysis

The obtained data are presented as mean values ± standard deviation (SD). Statistically significant differences were determined via a one-way analysis of variance (ANOVA) followed by Tukey’s post hoc test for multiple comparisons (GraphPad Prism version 5.03, San Diego, CA, USA). Probability values (*p*) ≤ 0.05 were considered to be statistically significant.

## 3. Results

### 3.1. Serum Biochemical Changes

Serum activities of ALT, AST, and γ-GT in control animals were within the normal physiological range, and a single Lyc administration did not produce notable changes (Table 1). On the other hand, a single LPS injection caused a marked, statistically significant elevation in ALT and AST compared to the control. Co-treatment with Lyc prevented an increase in ALT and AST activities compared with LPS alone, although values remained above control levels (Table 1). γ-GT activity was found to be significantly increased after LPS administration, and the application of Lyc had almost no impact on its activity (Table 1). Total and direct bilirubin concentrations showed minimal variation between groups, remaining within physiological limits in all groups (Table 1).

### 3.2. Tissue Oxidative Stress Damage Parameters

Exposure of rats to LPS led to a significant increase in XO and MPO activity, which was followed by a significant increase in tissue TBARS and PCC (Table 2). Application of Lyc in combination with LPS significantly prevented an increase in both XO and MPO activities, as well as in TBARS generation (Table 2). However, the PCC in the LPS + Lyc group remained significantly increased compared to the control animals (Table 2).

The activity of the studied antioxidant enzymes (CAT and SOD) was found to be significantly decreased in the livers of rats exposed to LPS only (Table 2). In the case of rats treated with LPS + Lyc, the activity of CAT and SOD was increased compared to the LPS-treated rats; however, activity in the case of SOD was still significantly lower than in the control group (Table 2).

### 3.3. Nitric Oxide Signaling Pathway

**Figure 1:**
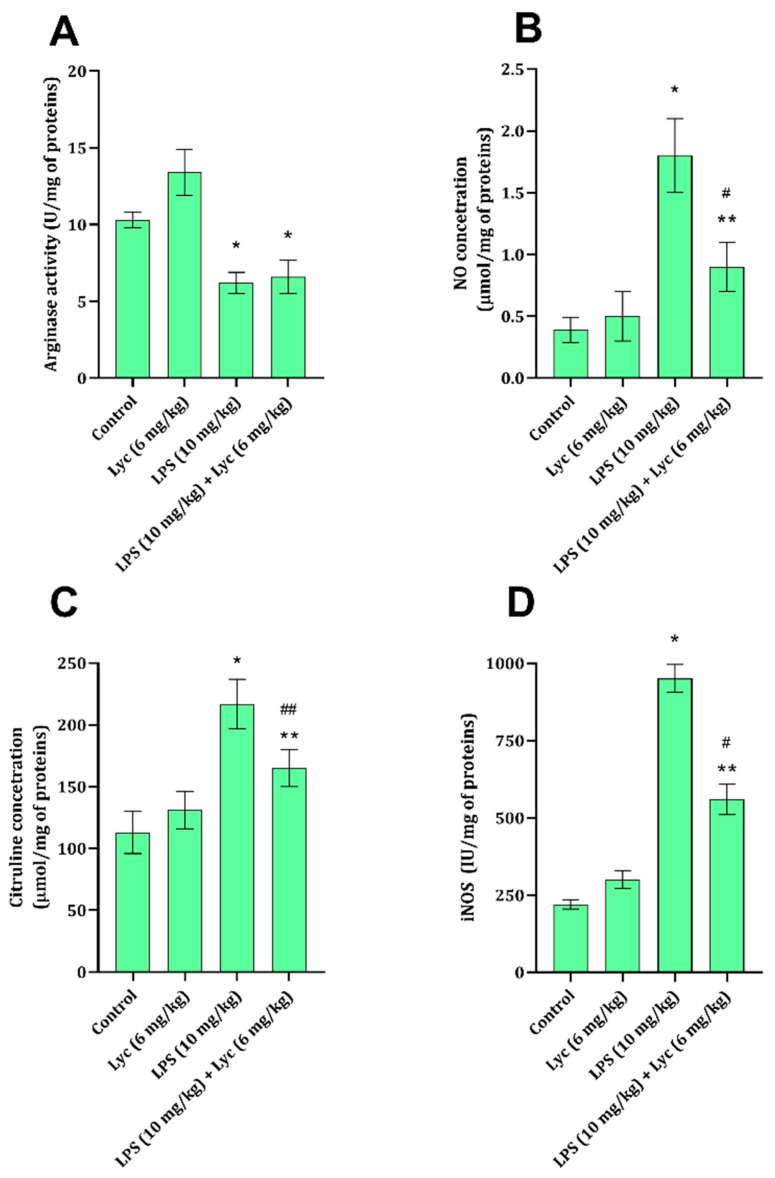
Arginase activity (**A**), NO concentration (**B**), citrulline concentration (**C**), and iNOS activity (**D**) in rat liver tissue belonging to different experimental groups. Data are shown as mean ± SD (n = 6). One-way ANOVA, followed by Tukey’s post hoc test, * *p* < 0.001, ** *p* < 0.01 vs. control; ^#^ *p* < 0.001, ^##^ *p* < 0.01 vs. LPS-treated animals. LPS—lipopolysaccharide; Lyc—lycopene.

Application of LPS led to a statistically significant decrease in liver tissue arginase activity and a significant increase in NO and citrulline concentrations and iNOS activity (Figure 1A–D). At the same time, application of Lyc together with LPS did not produce a significant decrease in NO and citrulline concentrations and iNOS activity (Figure 1C,D), but had no impact on arginase activity (Figure 1A). When rats were exposed to Lyc on its own, no changes in the studied parameters related to the NO signaling pathway were noted (Figure 1A–D).

### 3.4. Tissue Glutathione Cycle Parameters

**Figure 2:**
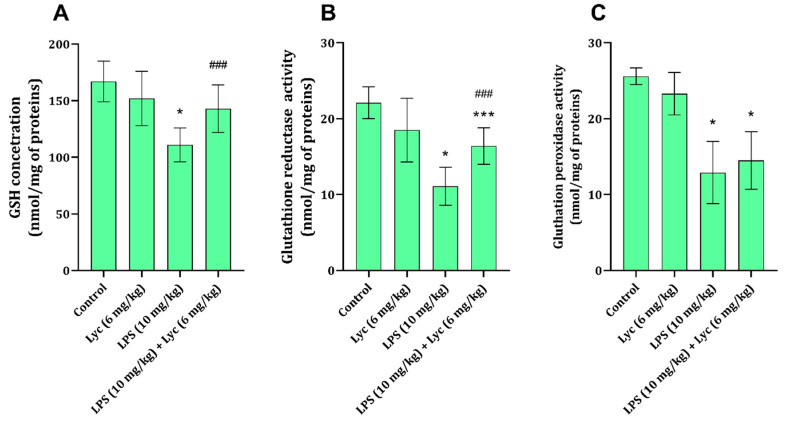
Rat liver tissue GSH (**A**), GR (**B**), and GPx (**C**) activities obtained from different experimental groups. Data are shown as mean ± SD (n = 6). One-way ANOVA, followed by Tukey’s post hoc test, * *p* < 0.001, *** *p* < 0.05 vs. control; ^###^ *p* < 0.05 vs. LPS-treated animals. LPS—lipopolysaccharide; Lyc—lycopene.

Liver tissue GSH content was found to be significantly decreased 12 h after LPS application (Figure 2A). The amount of GSH in animals that received Lyc together with LPS was significantly higher (*p* < 0.05) than the amount found in animals receiving only LPS (Figure 2A). At the same time, the activities of GR and GPx in rats exposed only to LPS were found to be significantly decreased compared to the control group of healthy animals (Figure 2B,C). Application of Lyc only prevented an LPS-associated decrease in GR activity (Figure 2B), but it did not affect GPx activity (Figure 2C).

### 3.5. Tissue Apoptosis and Inflammation-Associated Parameters

The liver tissue activity of DNases I and II (IU/g of proteins) in rats treated with LPS was found to be significantly higher than the activities of both DNases in the liver of control animals (Table 3). Application of Lyc (6 mg/kg) and LPS also increased the activity of the two mentioned enzymes; however, the extent of the increase was significantly lower than in the group that received only LPS (Table 3). Animals exposed to LPS had statistically significantly increased liver NF-κB content, as well as higher concentrations of both IL-6 and TNF-α, compared to healthy animals (Table 3). Application of Lyc together with LPS prevented an increase in NF-κB content compared to the LPS-treated rats, but the content was still significantly higher than in the control group (Table 3). The two studied cytokines, IL-6 and TNF-α, were found to be decreased in the group of rats treated with Lyc and LPS when compared to the LPS group (Table 3). The liver tissue Nrf-2 content was found to be significantly decreased in animals treated with LPS when compared to the control group values (Table 3). The combined application of Lyc and LPS failed to prevent this significant decrease in Nrf-2 levels, with the concentrations approximately equal to those of the LPS-treated animals (Table 3).

## 4. Discussion

Sepsis and endotoxemia, mimicked in the present study by the application of LPS, are a worldwide cause of death in different patient populations [1]. The amount of circulating LPS is directly associated with a lethal outcome [1], and the application of Lyc has previously been found to decrease circulating LPS levels [6]. In experimental conditions, high-dose LPS leads to hepatocellular necrosis and pronounced inflammation. This immune system reaction and liver damage are associated with massive cell damage (cytolysis), which is followed by the release of a number of enzymes (ALT and AST) into the bloodstream [29]. The ability of Lyc to partially reduce serum ALT and AST has been confirmed by the results of the present study (Table 1) and by the results of some previous studies as well [30,31]. Additionally, LPS application led to an increase in serum γ-GT activity, a very specific enzyme located in bile duct endothelial cells and considered a specific marker of liver damage [32]. Co-application of Lyc with LPS did not change the activity of this enzyme, suggesting that Lyc cannot protect the bile duct endothelium.

Increased ROS can be the consequence of either increased production through several systems (here, XO and MPO were studied) or due to a decrease in enzymes responsible for their removal (CAT and SOD). This imbalance is evident in the case of LPS-induced liver damage, which is tightly associated with the activation of TLR-4 and an increase in tissue XO [33] or MPO [34] activities, as confirmed by the results of this study (Table 2). Both of these enzymes are able to generate different ROS forms [35], causing damage to cell building molecules, i.e., lipids and proteins. Examined liver tissue showed a marked increase in TBARS and PCC content (Table 2), indicating damage to the lipid and protein cell structures, respectively. Also, a disturbance in antioxidant defense enzymes, CAT and SOD, was observed in rats exposed to LPS, which might be due to high ROS production and thus demand for their removal, as well as due to cell damage and inability to synthetize the mentioned enzymes (Table 2). One of the mechanisms proposed for Lyc and other carotenoids involves their ability to scavenge ROS via an electron-transfer reaction [11,36], thereby preventing oxidative damage. We speculate that the Lyc in this case, also via the suggested mechanism of ROS mediation, prevented lipid and protein damage. In an acute mouse liver damage model, Lyc application, in doses ranging from 100 to 10 mg/kg, produced an increase in CAT [37]. At the same time, in a prolonged experiment, chronic Lyc application led to a significant increase in liver SOD activity, which was diminished by nonalcoholic fatty liver disease [38] or bile duct ligation [39]. Such effects were observed in the present study as well; however, the effect on CAT activity was more pronounced than on that of the SOD. This prevention of cell structural molecules further impacts the liver tissue state and response in a state of sepsis.

Nitric oxide is one of the gaseous neurotransmitters involved in different signaling cascades, both intracellular and extracellular. In the cases of tissue damage, inflammation, or generally under stress, it is largely produced through inducible nitric oxide synthase (iNOS) from arginine [40]. Under different conditions, when arginase activity prevails in cells, arginine is degraded to urea and ornithine; thus, the amount of arginine is limited for iNOS [41]. The overproduction of NO by iNOS, such as seen after exposure to LPS (Figure 1B,D), has numerous devastating consequences on cells. In its reaction with hydroxyl radicals, RNS are formed, which further exacerbate cell damage [42] and inhibit arginase activity [43]. Also, an increase in NO is marked by ATP depletion, ultimately contributing to hepatocellular apoptosis or necrosis [44], thus promoting systemic inflammatory progression and multiorgan failure. The role of Lyc has been previously suggested to involve NO signaling pathways, and this has been demonstrated in both animal [31] and human studies [45].

In the state of endotoxemia, liver tissue GSH levels are known to be decreased, as is evident from the results of the present study (Figure 2A), either due to an increase in ROS or due to a decrease in GSH synthesis [46]. The same also applies to GR activity, which is involved in the conversion from oxidised glutathione to GSH (Figure 2B), thus maintaining cell GSH levels. Additionally, a decrease in one of the enzymes in the glutathione system, GPx, is associated with an increase in ROS and damage to macromolecular structures [47]. These mechanisms were confirmed by the results of the present study, indicating that LPS application decreases liver tissue GSH and GPx (Figure 2A,B), which can be directly associated with an increase in TBARS and PCC (Table 2). The ability of Lyc to prevent LPS-induced decrease in GSH and disturbance in enzymes metabolising GSH has been partially proven (Figure 2A–C), and some of these effects have been proven in different liver-damaging models [31,48]. Although the GSH content and GR activity remained slightly affected by LPS application in the presence of Lyc, the activity of GPx remained decreased (Figure 2C). Interestingly, GPx activity consequently impacts TBARS and PCC [47], and one would expect that the oxidative damage parameters were increased in the LPS + Lyc group; however, they appeared lower than in the LPS group (Table 2). The reason for such findings might be explained through the effects of Lyc on other enzymes generating ROS (Table 2), NO signalization (Figure 1), and its direct ability to interact with ROS [36].

Exposure to LPS and the development of sepsis cause oxidative damage to cell structures, and breach of oxidative defences leads to programmed cell death, genome destruction, and DNA leakage into circulation [3]. The role of the studied DNases, each under different cell pH conditions, is to break DNA strands during apoptosis and prevent DNA from exiting the cell and further activate and enhance the immune system response [49]. As one might expect, the activity of the two enzymes was found to increase in animals exposed to LPS only (Table 3), indicating that the liver cells undergo apoptosis. The application of Lyc significantly prevented this increase in DNase activities, pointing to the fact that this carotenoid compound prevents the apoptosis process (Table 3). Also, a significant increase in DNase activities in the Lyc + LPS group of rats is not the consequence of the prevention of damage to cellular building molecules, lipids, and proteins (Table 2), as indicated by lower liver cell-damage enzyme activities in the serum (Table 1).

The immune system reaction that occurs after LPS application involves the generation of a plethora of proinflammatory cytokines and chemokines, mainly mediated via TLR4 activation [4] and further NF-κB translocation to the nucleus [3]. The dysfunction in the innate immune system activity has been, apart from TLR4 activation, also associated with a decrease in GSH [50] and a decrease in antioxidative defenses [3]. Increased NF-κB content and cytokines TNF-α and IL-6 were found in rats exposed to LPS (Table 3). Keeping in mind the explained mechanism of NF-kB activation, it is not unexpected to be upregulated in animals with sepsis. On the other hand, cytokines TNF-α and IL-6 are known to be affected (upregulated and downregulated) by different signaling pathways, one of which is Nrf-2, whose activation causes a decrease in these cytokines [3].

Application of Lyc with LPS (Lyc + LPS group) partially prevented an increase in NF-κB and almost completely prevented an increase in TNF-α and IL-6 concentrations (Table 3). Previous studies also found that Lyc decreases the extent of inflammatory reaction by inhibiting liver TLR4 signaling and reducing serum TNF-α, IL-6, and IL-1β, linking its ability to reduce LPS serum levels directly [6], similar to results that indicate that carotenoids can decrease TNF-α and IL-6 in inflamed colonic tissue [13]. As suggested, the mechanisms modulating cytokine production are not only based on NF-κB, and others might be involved in the activity associated with Lyc application.

One of the mechanisms explaining massive damage and inadequate response to ROS is a decrease in sumoylation of Nrf2 via direct inhibition of enzymes related to its stability [51]. Nrf2 is crucial in cell defence against ROS, and in stressful events, it translocates from cytoplasm to the nucleus, upregulating genes involved in defence and survival [52]. This being said, it is not surprising that LPS application decreases Nrf2 liver tissue only 12 h after injection (Table 3). Interestingly, Lyc application failed to reduce Nrf2 content, which remained almost identical to that in the LPS group (Table 3). Up to now, studies have shown that the metabolites of Lyc are responsible for Nrf2 activation and an increase in Nrf2-associated enzyme activities [53,54,55]. It is worth mentioning that the potential reason for this is that, in the mentioned studies, either the cells had a higher metabolic potential (cancer cells) [53], or the effects were observed in the kidneys [54] in the presence of a kidney-damaging substance. This study showed the effects of Lyc in liver tissue involved another liver damage model [55] and cannot be fully correlated with the present one, which involves systemic and local organ damage [6].

One of the suggested mechanisms through which Lyc might impact liver function, improve recovery, and prevent damage is by acting on gastrointestinal microorganisms, including both bacteria and fungi (microbiota) [56]. The role of the microbiota in non-alcoholic fatty liver disease has been shown both in animals and humans [57], and the role of the gut–liver axis during sepsis has been under investigation as well [58]. Application of Lyc during 8 weeks led to an altered mouse microbiota, leading to a decrease in “destructive’’ bacteria (e.g., *Firmicutes*, *Lachnospiraceae_NK4A136_group*, *Desulfovibrio*, and *Alistipes*) and an increase in *Allobaculum*, a species known for its ability to produce short-chain fatty acids [59]. These results indicate that Lyc might impact liver damage during sepsis by affecting the gut–liver axis as well. The role of Lyc on chicken liver cell damage associated with fungal toxins (fumonisins) has been investigated recently [60]. It was concluded that in the mentioned in vitro model, Lyc prevents mitochondrial activation and ROS accumulation, and that its mitophagy-modulating activity is mediated via sirutin 3 pathway, or to be more precise, via FOXO3(Forkhead Box O3)-BNIP3L (BCL2/adenovirus E1B 19-kDa-interacting protein 3-like) [60].

## 5. Conclusions

The findings of this study demonstrated lycopene’s partial hepatoprotective effects in the LPS-induced liver injury model in rats. Lycopene administration attenuated hepatocellular enzyme release, reduced oxidative stress damage molecules, and partially affected NF-κB-mediated proinflammatory cytokine production. It also restored GSH levels and glutathione reductase activity while preventing apoptosis, as indicated by DNase I/II activities. However, lycopene had limited effects on GPx activity, nitric oxide/iNOS signaling, and Nrf2 expression, suggesting that some oxidative and inflammatory pathways remain unaffected by its presence. Further studies need to be designed and conducted in order to clarify tissue-specific mechanisms and optimize dosing strategies for translational applications. This should be of great importance, since lycopene has been recognized as a potential drug (or supplement drug) candidate for many inflammation-based disorders.

## Figures and Tables

**Table 1:** Serum biochemical parameters obtained from rats belonging to different experimental groups.

Serum Parameter	Control	Lyc (6 mg/kg)	LPS (10 mg/kg)	LPS (10 mg/kg) + Lyc (6 mg/kg)
ALT (U/L)	49 ± 5.2	51.1 ± 4.7	212 ± 39.1 *	145 ± 22.8 ^#,^**
AST (U/L)	138 ± 17.1	152 ± 21.8	753 ± 158.5 *	499 ± 187 ^##,^*
γ-GT (U/L)	2.4 ± 0.12	2.2 ± 0.18	3.2 ± 0.4 **	3.2 ± 0.6 **
Total bilirubin (μmol/L)	1.9 ± 0.33	1.9 ± 0.6	2.1 ± 0.6	2.25 ± 0.5
Direct bilirubin (μmol/L)	0.53± 0.13	0.39 ± 0.05	0.48 ± 0.13	0.51 ± 0.19

Data are shown as mean ± SD (n = 6). One-way ANOVA, followed by Tukey’s post hoc test, * *p* < 0.001, ** *p* < 0.01 vs. control; ^#^ *p* < 0.001, ^##^ *p* < 0.01 vs. LPS-treated animals. ALT—alanine aminotransferase; AST—aspartate aminotransferase; LPS—lipopolysaccharide; Lyc—lycopene; γ-GT—gamma-glutamyltransferase.

**Table 2:** Liver tissue parameters associated with ROS generation and damage, as well as enzymes associated with antioxidant defense, obtained from rats belonging to different experimental groups.

Serum Parameter	Control	Lyc (6 mg/kg)	LPS (10 mg/kg)	LPS (10 mg/kg) + Lyc (6 mg/kg)
XO (IU/mg of proteins)	0.98 ± 0.26	1.1 ± 0.17	2.24 ± 0.41 *	1.21 ± 0.4 ^#^
MPO (OD/mg of proteins)	80 ± 7.8	92 ± 11.5	162 ± 15.9 *	88 ± 17 ^#^
TBARS (μmol/ mg of proteins)	1.7 ± 0.33	1.7 ± 0.6	3.1 ± 0.6 *	1.55 ± 0.5 ^#^
PCC (μmol/ mg of proteins)	5.9 ± 1.8	8.4 ± 5.1	27.8 ± 6.1 *	19.9 ± 5.8 ^##,^*
CAT (IU/mg of proteins)	1.17 ± 0.1	1.24 ± 0.2	0.52 ± 0.1 *	1.02 ± 0.2 ^#^
SOD (% decrease)	100 ± 7.4	102 ± 5.3	54.9 ± 8.5 *	82.3 ± 6.1 ^##,^**

Data are shown as mean ± SD (n = 6). One-way ANOVA, followed by Tukey’s post hoc test, * *p* < 0.001, ** *p* < 0.01 vs. control; ^#^ *p* < 0.001, ^##^ *p* < 0.01 vs. LPS-treated animals. LPS—lipopolysaccharide; Lyc—lycopene; MPO—myeloperoxidase; PCC—protein carbonyl content; TBARS—thiobarbituric acid reactive substances; XO—xanthine oxidase.

**Table 3:** Liver tissue apoptosis and inflammation-associated parameters obtained from rats belonging to different experimental groups.

Serum Parameter	Control	Lyc (6 mg/kg)	LPS (10 mg/kg)	LPS (10 mg/kg) + Lyc (6 mg/kg)
DNase I (IU/g of proteins)	4.1 ± 0.6	4.6 ± 0.2	9.5 ± 0.7 *	5.6 ± 0.8 ^#,^***
DNase II (IU/g of proteins)	5.6 ± 0.5	5.1 ± 0.7	15.6 ± 4.4 *	7.9 ± 2.5 ^##,^**
NF-κB (pg/mg of proteins)	29.9 ± 11.5	33.7 ± 7.8	221.5 ± 33.8 *	145.2 ± 19.4 ^#,^*
IL-6 (pg/mg of proteins)	1.3 ± 0.8	1.2 ± 0.2	6.5 ± 1.2 *	3.4 ± 1.1 ^#,^***
TNF-α (pg/mg of proteins)	32.5 ± 12.1	31.4 ± 8.1	105.5 ± 16.5 *	43.9 ± 12.5 ^#^
Nrf2 (pg/mg of proteins)	400 ± 48	385 ± 26	256 ± 27 *	298 ± 14 *

Data are shown as mean ± SD (n = 6). One-way ANOVA, followed by Tukey’s post hoc test, * *p* < 0.001, ** *p* < 0.01, *** *p* < 0.05 vs. control; ^#^ *p* < 0.001, ^##^ *p* < 0.01 vs. LPS-treated animals. DNase I—deoxyribonuclease I DNase II—deoxyribonuclease II; IL-6—interleukin-6; LPS—lipopolysaccharide; Lyc—lycopene; NF-κB—nuclear factor kappa-light-chain-enhancer of activated B cells; Nrf2—nuclear factor erythroid 2-related factor 2; TNF-α—tumor necrosis factor alpha.

## Data Availability

The original contributions presented in this study are included in the article. The data are available upon reasonable request from the corresponding author.

## Abbreviations

The following abbreviations are used in this manuscript:

ALT	Alanine aminotransferase.
Arg	Arginase.
AST	Aspartate aminotransferase.
Cit	Citrulline.
DNase I/II	Deoxyribonuclease I/II.
γ-GT	Gamma-glutamyltransferase.
GSH	Reduced glutathione.
GPx	Glutathione peroxidase.
GR	Glutathione reductase.
IL-6	Interleukin-6.
iNOS	Inducible nitric oxide synthase.
Lyc	Lycopene.
LPS	Lipopolysaccharide.
MPO	Myeloperoxidase.
NF-κB	Nuclear factor kappa-light-chain-enhancer of activated B cells.
Nrf2	Nuclear factor erythroid 2–related factor 2.
NO	Nitric oxide.
PCC	Protein carbonyl content.
TBARS	Thiobarbituric acid reactive substances.
TNF-α	Tumor necrosis factor alpha.
XO	Xanthine oxidase.
